# Optimization and Entropy Production: Application to Carnot-Like Refrigeration Machines

**DOI:** 10.3390/e20120953

**Published:** 2018-12-11

**Authors:** Camelia Stanciu, Michel Feidt, Monica Costea, Dorin Stanciu

**Affiliations:** 1 Department of Engineering Thermodynamics, University POLITEHNICA of Bucharest, 060042 Bucharest, Romania; 2 LEMTA, URA CNRS 7563, University of Lorraine, 54518 Vandoeuvre-lès-Nancy, France

**Keywords:** Carnot machine, refrigeration, entropy production, thermodynamics, optimization

## Abstract

Several optimization models of irreversible reverse cycle machines have been developed based on different optimization criteria in the literature, most of them using linear heat transfer laws at the source and sink. This raises the issue how close to actual operation conditions they are, since the heat transfer law on the phase-change processes is dependent on Δ*T*^3^. This paper addresses this issue by proposing a general model for study and optimization of thermal machines with two heat reservoirs applied to a Carnot-like refrigerator, with non-linear heat transfer laws and internal and external irreversibility. The optimization was performed using First and Second Law of Thermodynamics and the Lagrange multipliers method. Thus, several constraints were imposed to the system, also different objective functions were considered, allowing finding the optimum operating conditions, as well as the limited variation ranges of the system parameters. Results show that the nature of the heat transfer laws affects the optimum values of system parameters for obtaining maximum performances and also their magnitude. Sensitivity studies with respect to system several parameters are presented. The results contribute to the understanding of the system limits in operation under different constraints and allow choosing the most convenient variables in given circumstances.

## 1. Introduction

The second half of the last century saw the emergence of a new branch of Thermodynamics, called Thermodynamics in Finite Time, due to the process duration that was considered by the heat transfer rate [1,2]. The corresponding approach emphasized a maximum power regime [3] for an endo-reversible, but exo-irreversible engine. The engine efficiency at maximum power regime is less than the Carnot cycle one, and it introduced the particular form of the “nice radical”.

Since then, quite a lot of works analyzing the exo-irreversible engines have been developed [4,5]. Generally, they have considered only the external irreversibility of the thermal machine, which is due to the heat transfer at finite temperature difference between the sources and the working fluid. Also, mainly the linear form of the heat transfer law was used.

The same approach applied to the study of heat pumps shows no more optimum for the useful effect (heat flow delivered at the hot end), because the temperature of the working fluid has no upper limit imposed on the hot end [6]. The same happens for the refrigerating machine, where there is no lower limit to the cold end (as for thermal engine).

To the first reported work (to our knowledge) [6], other papers followed [7,8,9] dealing with heat pump or refrigerating machine optimization, but introducing an additional constraint (mostly mechanical power or useful effect imposed). The optimization is not reserved only to vapor mechanical compression machines, but it has been extended since that time to the trithermal and quadrithermal ones [10,11,12]. However, one notes that most of the reported studies deal with thermostats at sources and sinks and linear heat transfer laws.

Proposals have been made to extend the analysis by using other forms of the heat transfer law [13,14,15]. Further progress has been made by introducing an irreversibility factor (ratio), most often constant [16], that accounts for the internal irreversibility of the machine (endo-irreversibility). A step further [17] represents the internal irreversibility considered in the model as internal entropy production rate, ${\dot{S}}_{i}$. This method is preferred to the one of ratios, as we still see in recent work [18]. It provides a more general approach compared to the use of the irreversibility factor [16,19,20]. Also, it aims the entropic analysis of machines and processes and is more and more important as an instrument getting modeling closer to real operation [21,22,23,24,25,26,27].

The present work proposes a general model of study and optimization of irreversible Carnot-like refrigerating machines and extends the previous studies [28,29,30,31]. In terms of optimization, it is well known that several objective functions can be chosen: maximum of coefficient of performance (COP), minimum of energy consumption, minimum of total entropy production, or economic objectives [32]. Environmental concerns have also become predominant [33], and recently led to the introduction of a new objective called Ecological COP (ECOP) [34]. Two objective functions will be considered here for each of the two studied cases: *maximum refrigeration load* and *minimum total entropy production rate* when COP is imposed, respectively, *maximum COP* and *minimum total entropy production rate,* for imposed refrigeration load. We limit the presentation only to these cases as the main target is to show how the method is applied and what the relevance of the obtained results is, but it could be extended and applied for other cases of imposed constraints, such as consumed mechanical power or heat transfer restricted at the hot source, for which objective functions could be sought.

The mathematical approach includes the most general laws of science, namely the First and Second Laws of Thermodynamics. The equations of heat transfer at the hot and cold side of the machine are modeled by non-linear functions of the temperature difference between the reservoirs and the cycle working fluid. The internal irreversibility is introduced by the method of internal entropy production rate, above mentioned. Three variation laws with temperature for the internal entropy production are considered in an attempt to approach analytical treatment of actual operation.

The purpose of this work is to study the influence of the non-linearity of the heat transfer law on the performance. This will *extend the model validity* beyond the convective and radiative heat transfer laws [13]. The results highlight the existence of optimal operating regimes of the reverse cycle machine subjected to dimensional and operating constraints. The sensitivity study with respect to the model parameters provides interesting results related to the *limitation of the variation range of the variables* of the model, and *different operating regime* of the refrigeration machine. The new and important results reported here are presently extended by the study of other cases of imposed constraints.

## 2. Materials and Methods

### 2.1. Proposed Application Study

**Figure 1:**
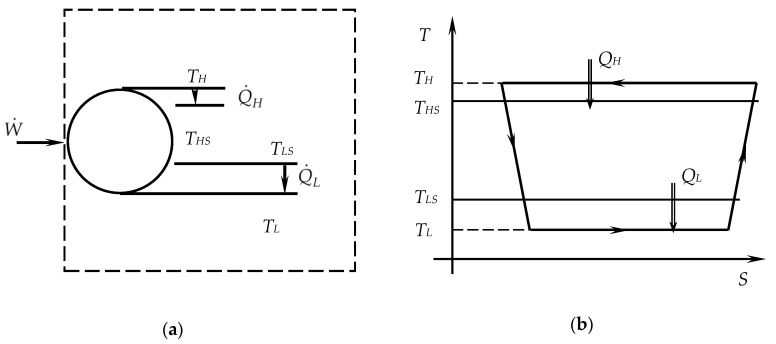
Irreversible Carnot-like refrigeration machine: (**a**) Scheme; (**b**) Irreversible cycle in *T*-*S* diagram.

The model of study and optimization of Carnot-like machines is applied here to an irreversible refrigeration machine. Its architecture and the corresponding irreversible cycle consisting of two isothermal processes and two adiabatic irreversible ones are illustrated in Figure 1. The irreversibility is present in the T-S diagram by the entropy production on the adiabatic processes (compression and expansion), which is mainly due to internal losses. On the isothermal processes, it is marked by the different heat transfer compared to the corresponding reversible processes (also more reduced at the source and larger at the sink).

Usually, the internal irreversibility of the machine, introduced in the model as a parameter, is represented by the corresponding internal entropy production term, ${\dot{S}}_{i}$. It takes into account the internal irreversibility generated on each cycle process, summing the throttling losses, friction ones, etc. To this one an external irreversibility is added, being generated by finite temperature heat transfer processes between the working fluid and the heat reservoirs. 

As shown in Figure 1, the two heat reservoirs are thermostats, of constant temperatures *T_HS_* and *T_LS_*, the first one being actually the environment. The refrigeration machine is considered operating in steady state regime.

### 2.2. Mathematical Model

The model of study and optimization of reverse cycle machines is developed by using the most general laws in Thermodynamics, the First and Second Law, to which generalized forms of heat transfer laws applied to the source and sink are added.

By taking account of the sign convention adopted here, that considers the heat transfer rate positive when entering the cycle, and negative for leaving it, and the consumed work transfer rate (power request) as negative, the expression of the First Law of Thermodynamics can be written as:

(1) $$\dot{W}={\dot{Q}}_{H}+{\dot{Q}}_{L},$$

with $\dot{W}$—mechanical power supplied; ${\dot{Q}}_{H}$—heat transfer rate rejected by the working fluid at the source (hot heat exchanger); ${\dot{Q}}_{L}$—refrigeration load.

When looking the machine irreversibility, the Second Law of Thermodynamics takes different expressions, as applied to two possible systems. Thus, once considering only the internal irreversibilities (endo-irreversible machine) by the internal entropy production term, it becomes:

(2) $$\frac{{\dot{Q}}_{H}}{T_{H}}+\frac{{\dot{Q}}_{L}}{T_{L}}+{\dot{S}}_{i}=0,$$

with *T_H_*—temperature of the working fluid at the machine hot temperature side; *T_L_* —temperature of the working fluid at the machine low temperature side; ${\dot{S}}_{i}$—ntropy production due to internal irreversibilities of the machine.

Then, when the whole system is considered (cycle and the two heat reservoirs), the total entropy production term appears in the Second Law expression:

(3) $$\frac{{\dot{Q}}_{H}}{T_{HS}}+\frac{{\dot{Q}}_{L}}{T_{LS}}+{\dot{S}}_{T}=0,$$

with *T_HS_*—source temperature at the machine hot part; *T_LS_*—source temperature at the machine cold part; ${\dot{S}}_{T}$—total entropy production (internal and external).

For the heat transfer rates present in the above equations, two forms of non-linear heat transfer laws between the heat reservoirs and working fluid are considered:

(4) $${\dot{Q}}_{H}=K_{H,gen}{\left(T_{HS}–T_{H}\right)}^{n_{H}}{\left(–1\right)}^{n_{H}–1}\;;\;{\dot{Q}}_{L}=K_{L,gen}{\left(T_{LS}–T_{L}\right)}^{n_{L}},$$

(5) $${\dot{Q}}_{H}=K_{H,gen}\left(T_{HS}^{n_{H}}–T_{H}^{n_{H}}\right)\;;\;{\dot{Q}}_{L}=K_{L,gen}\left(T_{LS}^{n_{L}}–T_{L}^{n_{L}}\right),$$

where *n_i_* is the exponent of the heat transfer law (*H*—hot sink or high temperature side, *L*—cold source or low temperature side).

In practical application these non-linear forms correspond to phase-change heat transfer (Equation (4), with *n_i_* = 3), respectively radiation heat transfer (Equation (5) with *n_i_* = 4). One note that making *n_i_* = 1, one gets the linear approximation, so called, convective heat transfer law or Newton’s law.

For the refrigeration machines performance evaluation, according to its definition, the coefficient of performance is:

(6) $$COP=\frac{{\dot{Q}}_{L}}{\left|\dot{W}\right|}.$$

By combining the two expressions of the Second Law, given by Equations (2) and (3), the total entropy production rate results as:

(7) $${\dot{S}}_{T}={\dot{S}}_{i}+{\dot{Q}}_{H}\left(\frac{1}{T_{H}}–\frac{1}{T_{HS}}\right)+{\dot{Q}}_{L}\left(\frac{1}{T_{L}}–\frac{1}{T_{LS}}\right).$$

The three positive terms contained in the above expression accounts for different type of irreversibility, namely (1) the internal one by ${\dot{S}}_{i}$, and (2) the external one due to finite temperature heat transfer processes between the heat reservoirs and working fluid.

Without insight in the internal irreversibility mechanism of machine processes, it is difficult to establish a variation law for the internal entropy production. Thus, in order to provide generality to the model, the following approaches were considered [35,36]:

(a) constant

(8) $${\dot{S}}_{i}=const,$$

(b) linear variation law with working fluid temperature difference:

(9) $${\dot{S}}_{i}=const\left(T_{H}–T_{L}\right),$$

(c) logarithmic variation law with working fluid temperature ratio:

(10) $${\dot{S}}_{i}=const\left(\ln\frac{T_{H}}{T_{L}}\right).$$

### 2.3. Dimensionless Model

The same concern for developing a general model for the optimization of refrigeration machines and easily adapted to different characteristics and constraints of the studied case, led to the adoption of the non-dimensional form of the equations. Therefore, the following terms and expressions will be further used:

- Dimensionless temperatures are expressed relative to the reference temperature *T_HS_* which is also the ambient one:
  (11) $$\theta_{SL}=\frac{T_{LS}}{T_{HS}}\;,\;\theta_{H}=\frac{T_{H}}{T_{HS}}\;,\;\theta_{L}=\frac{T_{L}}{T_{HS}}.$$
- Dimensionless energy fluxes are expressed relative to the product $\dot{m}c_{p}T_{HS}$, where $\dot{m}c_{p}$ is the heat rate capacity of the working gas corresponding to the end of the compression process:
  - at the sink or machine cold part (positive):
    (12a) $${\tilde{q}}_{L}=\frac{{\dot{Q}}_{L}}{\dot{m}c_{p}T_{HS}}=CTQ_{L,gen}{\left(\theta_{LS}–\theta_{L}\right)}^{n_{L}},$$
  - at the source or machine hot part (always negative)
    (12b) $${\tilde{q}}_{H}=\frac{{\dot{Q}}_{H}}{\dot{m}c_{p}T_{HS}}=CTQ_{H,gen}{\left(1–\theta_{H}\right)}^{n_{H}}{\left(–1\right)}^{n_{H}–1},$$
  - power supply:
    (13) $$\tilde{w}=\frac{\left|\dot{W}\right|}{\dot{m}c_{p}T_{HS}}=\frac{{\tilde{q}}_{L}}{COP}.$$
- Non-linear thermal conductances are expressed relative to the product $\dot{m}c_{p}T_{HS}^{-\left(n_{H}-1\right)}$:
  - total one:
    (14) $$CTQ_{T,gen}=\frac{{\left(UA\right)}_{T}T_{HS}^{\left(n_{H}–1\right)}}{\dot{m}c_{p}},$$
  - of the two heat exchangers:
    (15) $$CTQ_{L,gen}=\frac{{\left(UA\right)}_{L}T_{HS}^{\left(n_{H}–1\right)}}{\dot{m}c_{p}},$$
    (16) $$CTQ_{H,gen}=\frac{{\left(UA\right)}_{H}T_{HS}^{\left(n_{H}–1\right)}}{\dot{m}c_{p}}.$$
- Internal and total entropy production terms use the same heat rate capacity, $\dot{m}c_{p}$, as reference:
  (17) $${\tilde{s}}_{i}=\frac{{\dot{S}}_{i}}{\dot{m}c_{p}}\;,\;$$
  (18) $${\tilde{s}}_{T}=\frac{{\dot{S}}_{T}}{\dot{m}c_{p}}\;.$$
- The dimensionless constraint relative to a fixed total heat exchanger conductance will have as equivalent:
  (19) $$CTQ_{T,gen}=CTQ_{H,gen}+CTQ_{L,gen},$$
  which will be considered as optimization constraint finite parameter, while *CTQ_H,gen_* and *CTQ_L,gen_* are system optimization variables.

According to the adopted sign convention and the refrigeration machine scheme, the temperatures hierarchy is *T_L_* < *T_LS_* < *T_HS_* < *T_H_*, that imposes the following constraints:

(20) $$\theta_{L}<\theta_{LS}<\theta_{HS}<\theta_{H},\;\theta_{HS}=1,\;{\tilde{q}}_{H}<0\;,\;{\tilde{q}}_{L}>0\;,\;\tilde{w}<0\;,\;{\tilde{s}}_{i}>0.$$

These constraints will always be valid for any refrigerator being studied, regardless of the presence of any other restrictions imposed by the user or the machine design.

### 2.4. Optimization Method and Studied Cases

The dimensionless variables that will be used in the optimization procedure are *θ_L_* and *CTQ_L,gen_*, *θ_H_* and *CTQ_H,gen_*, while the considered parameters are: *θ_LS_*, *CTQ_T,gen_*, ${\tilde{s}}_{i}$, and the imposed performance characteristics.

The optimization is achieved for two cases where different performances are imposed. For a refrigeration machine, the most important performances are the *COP* and the refrigeration load.

The first studied case corresponds to an *imposed value of the COP*, together *with an imposed internal entropy production rate*. The objective functions could be: maximum refrigeration load, minimum total dissipation (minimum total entropy production), or minimum power consumption.

Among them, results will be reported here when the maximum refrigerating load is sought, respectively, minimum total entropy production rate.

The second optimization is done for an *imposed refrigerating load*, together *with an imposed internal entropy production rate*. Thus, other objective functions will be sought, such as maximum *COP*, minimum total dissipation, or minimum power consumption. Again, only the first two objective functions previously cited will be considered in this analysis, for the sake of comprehensible presentation but the method is similarly applied.

The optimization procedure uses the *Lagrange Multipliers Method* that will lead to a system of equations for each objective function, and its solution will provide optimal values of the control variables of the system, *θ_L_*, *CTQ_L,gen_*, *θ_H_*, *CTQ_H,gen_*, leading to the corresponding system performances achieved in the considered constraint operation. Thus, the Lagrangian function corresponding to the present study is expressed as:

(21) $$L=FO+\lambda_{1}C_{1}+\lambda_{2}C_{2},$$

where: *FO* is one of the considered objective functions stated above; *λ*_1_ and *λ*_2_ are the corresponding Lagrange multipliers; *C*_1_ and *C*_2_ are the problem constraints represented by the First Law of Thermodynamics including the imposed performance characteristic constraint and Second Law of Thermodynamics applied to the cycle including the internal entropy production restriction.

The optimal values of the model variables are obtained by solving the following system of equations:

(22) $$\frac{\partial L}{\partial var}{)}_{1\to n\_var}\;,\;\frac{\partial L}{\partial \lambda_{1}}=0\;,\;\frac{\partial L}{\partial \lambda_{2}}=0.$$

#### 2.4.1. Imposed Coefficient of Performance

The First Law of Thermodynamics combined with Equation (13) and Equations (12a)–(12b) that takes into account the expressions of generalized heat transfer laws provides:

(23) $$CTQ_{H,gen}{\left(1–\theta_{H}\right)}^{n_{H}}{\left(–1\right)}^{n_{H}–1}+CTQ_{L,gen}{\left(\theta_{LS}–\theta_{L}\right)}^{n_{L}}\frac{1+COP}{COP}=0.$$

By generalizing the above expression, the ***first constraint equation (C1)*** of the model results as:

(24) $$CTQ_{H,gen}f+CTQ_{L,gen}\frac{1+COP}{COP}\xi =0,$$

where the following notations of the new functions were used:

(25) $$f={\left(1–\theta_{H}\right)}^{n_{H}}{\left(–1\right)}^{n_{H}–1}\;\xi ={\left(\theta_{LS}–\theta_{L}\right)}^{n_{L}},$$

One notes that *f* is a function of *θ_H_* only, while *ξ* is a function of *θ_L_* only. The Second Law of Thermodynamics combined with Equations (12a)–(12b) and applied to the cycle gives:

(26) $$\frac{CTQ_{H,gen}{\left(1–\theta_{H}\right)}^{n_{H}}{\left(–1\right)}^{n_{H}–1}}{\theta_{H}}+\frac{CTQ_{L,gen}{\left(\theta_{LS}–\theta_{L}\right)}^{n_{L}}}{\theta_{L}}+{\tilde{s}}_{i}=0,$$

and considering the two function introduced by Equation (25), the ***second constraint (C2)*** of the system results as:

(27) $$CTQ_{H,gen}\frac{f}{\theta_{H}}+CTQ_{L,gen}\frac{\xi }{\theta_{L}}+g=0.$$

One notes that *g* is a function of both *θ_H_* and de *θ_L_* according to the three variation laws adopted for the entropy production rate dependence on temperature, Equations (8)–(10).

*1. Refrigeration Load as Objective Function*

The first objective function considered in the optimization procedure is the refrigeration load:

(28) $$FO={\tilde{q}}_{L}=CTQ_{L,gen}{\left(\theta_{LS}–\theta_{L}\right)}^{n_{L}}=CTQ_{L,gen}\xi .$$

The Lagrangian is expressed in this case as:

(29) $$CTQ_{L,gen}\xi +\lambda_{1}\left[\left(CTQ_{T,gen}–CTQ_{L,gen}\right)f+CTQ_{L,gen}\xi \left(1+COP\right)/COP\right]+\;+\lambda_{2}\left[\left(CTQ_{T,gen}–CTQ_{L,gen}\right)f/\theta_{H}+CTQ_{L,gen}\xi /\theta_{L}+g\right]=0.$$

The derivatives indicated in Equation (22) are calculated for the considered variables (*CTQ_L,gen_, θ_H_, θ_L_*) and the two multipliers (*λ*_1_*, λ*_2_), as follows:

(30) $$\frac{\partial L}{\partial CTQ_{L,gen}}=0:\;\;\xi +\lambda_{1}\left[–f+\xi \left(1+COP\right)/COP\right]+\lambda_{2}\left(–f/\theta_{H}+\xi /\theta_{L}\right)=0,$$

(31) $$\frac{\partial L}{\partial \theta_{H}}=0:\;\;\lambda_{1}\left(CTQ_{T,gen}–CTQ_{L,gen}\right)f’+\lambda_{2}\left[\left(CTQ_{T,gen}–CTQ_{L,gen}\right)\frac{\theta_{H}f’–f}{\theta_{H}^{2}}+g{’}_{H}\right]=0,$$

where *f’* and *g’_H_* are the derivatives of function *f* and *g* respectively, with respect to *θ_H_*;

(32) $$\frac{\partial L}{\partial \theta_{L}}=0:\;\;CTQ_{L,gen}\xi ’+\lambda_{1}CTQ_{L,gen}\frac{1+COP}{COP}\xi ’+\lambda_{2}{(CTQ}_{L,gen}\frac{\theta_{L}\xi ’–\xi }{\theta_{L}^{2}}+g{’}_{L})=0,$$

where *ξ’* and *g’_L_* are the derivatives of function *ξ* and *g* respectively, with respect to *θ_L_*;

(33) $$\frac{\partial L}{\partial \lambda_{1}}=0:\;\;\;\left(CTQ_{T,gen}–CTQ_{L,gen}\right)f+CTQ_{L,gen}\frac{1+COP}{COP}\xi =0,$$

(34) $$\frac{\partial L}{\partial \lambda_{2}}=0:\;\;\;\left(CTQ_{T,gen}–CTQ_{L,gen}\right)\frac{f}{\theta_{H}}+CTQ_{L,gen}\frac{\xi }{\theta_{L}}+g=0.$$

By combining Equations (30)–(32) and after terms rearrangement one equation results that associated with Equations (33), (34) and (19) leads to a system that is solved by Newton-Raphson method with respect to *CTQ_L,gen_, θ_H_* and *θ_L_*, since *CTQ_H,gen_* is expressed by the last equation of the system.

(35) $$\frac{f^{2}}{\theta_{H}^{2}f’}-\frac{f{g^{\prime }}_{H}}{CTQ_{H,gen}f^{\prime }}=\frac{\xi^{2}}{\theta_{L}^{2}\xi ’}-\frac{\xi {g^{\prime }}_{L}}{CTQ_{L,gen}\xi^{\prime }}\;CTQ_{H,gen}f+\frac{1+COP}{COP}CTQ_{L,gen}\xi =0\;CTQ_{H,gen}\frac{f}{\theta_{H}}+CTQ_{L,gen}\frac{\xi }{\theta_{L}}+g=0\;CTQ_{H,gen}=CTQ_{T,gen}-CTQ_{L,gen}$$

Note that the system has to be solved for each of the three non-dimension functions *g* corresponding to the internal entropy production rate, Equations (8)–(10).

*2. Total Entropy Production Rate as Objective Function*

One considers here a second objective function, the total entropy production rate, expressed as:

(36) $$FO={\tilde{s}}_{T}=–{\tilde{q}}_{H}–\frac{{\tilde{q}}_{L}}{\theta_{LS}}=–CTQ_{H,gen}f–CTQ_{L,gen}\frac{\xi }{\theta_{LS}}.$$

The Lagrangian becomes in this case:

(37) $$–\left(CTQ_{T,gen}–CTQ_{L,gen}\right)f–CTQ_{L,gen}\frac{\xi }{\theta_{LS}}+\;+\lambda_{1}\left[\left(CTQ_{T,gen}–CTQ_{L,gen}\right)f+CTQ_{L,gen}\xi \left(1+COP\right)/COP\right]+\;+\lambda_{2}\left[\left(CTQ_{T,gen}–CTQ_{L,gen}\right)f/\theta_{H}+CTQ_{L,gen}\xi /\theta_{L}+g\right]=0.$$

The derivatives corresponding to the three variables (*CTQ_L,gen_, θ_H_, θ_L_*) and the two multipliers (*λ*_1_*, λ*_2_) are:

(38) $$\frac{\partial L}{\partial CTQ_{L,gen}}=0:\;\;\;f–\frac{\xi }{\theta_{LS}}+\lambda_{1}(–f+\frac{1+COP}{COP}\xi )+\lambda_{2}(–\frac{f}{\theta_{H}}+\frac{\xi }{\theta_{L}})=0,$$

(39) $$\frac{\partial L}{\partial \theta_{H}}=0:\;\;\;\left(CTQ_{T,gen}–CTQ_{L,gen}\right)f’\left(\lambda_{1}–1\right)+\lambda_{2}[\left(CTQ_{T,gen}–CTQ_{L,gen}\right)\frac{\theta_{H}f’–f}{\theta_{H}^{2}}+g{’}_{H}]=0,$$

(40) $$\frac{\partial L}{\partial \theta_{L}}=0:\;\;\;-\frac{CTQ_{L,gen}\xi ’}{\theta_{SL}}+\lambda_{1}CTQ_{L,gen}\frac{1+COP}{COP}\xi ’+\lambda_{2}[CTQ_{L,gen}\frac{\theta_{L}\xi ’–\xi }{\theta_{L}^{2}}+g{’}_{L}]=0,$$

where *f’*, *g’_H_*, *ξ’* and *g’_L_* have the same meaning as in Equations (31) and (32):

(41) $$\frac{\partial L}{\partial \lambda_{1}}=0:\;\;\;\left(CTQ_{T,gen}–CTQ_{L,gen}\right)f+CTQ_{L,gen}\frac{1+COP}{COP}\xi =0,$$

(42) $$\frac{\partial L}{\partial \lambda_{2}}=0:\;\;\;\left(CTQ_{T,gen}–CTQ_{L,gen}\right)\frac{f}{\theta_{H}}+CTQ_{L,gen}\frac{\xi }{\theta_{L}}+g=0.$$

Similar to the previous case, the corresponding equation system results as:

(43) $$\frac{f^{2}}{\theta_{H}^{2}f’}-\frac{f{g^{\prime }}_{H}}{CTQ_{H,gen}f^{\prime }}=\frac{\xi^{2}}{\theta_{L}^{2}\xi ’}-\frac{\xi {g^{\prime }}_{L}}{CTQ_{L,gen}\xi^{\prime }}\;CTQ_{H,gen}f+\frac{1+COP}{COP}CTQ_{L,gen}\xi =0\;CTQ_{H,gen}\frac{f}{\theta_{H}}+CTQ_{L,gen}\frac{\xi }{\theta_{L}}+g=0\;CTQ_{H,gen}=CTQ_{T,gen}-CTQ_{L,gen}$$

The comparison of the systems from Equations (35) and (43) shows that they are identical, meaning that no matter the objective function is considered, the same system of equations results. Thus, the optima of the two objective functions (maxima and minima, respectively) correspond to the same values of the model variables resulting from the solution of the equation system. The interpretation of this result lies on the fact that the total entropy production given by Equation (3) and written in non-dimensional form becomes:

(44) $${\tilde{s}}_{T}=-{\tilde{q}}_{H}-\frac{{\tilde{q}}_{L}}{\theta_{LS}}.$$

By considering Equation (1) in non-dimensional form and Equation (13), Equation (44) is re-written as:

(45) $${\tilde{s}}_{T}=-{\tilde{q}}_{L}\left(\frac{1}{COP}-1+\frac{1}{\theta_{LS}}\right).$$

As *COP* and *θ_SL_* are imposed for the system operation, the optimum for *q̃_L_* will lead to optimum for *s̃_T_* in the same time. More specific, maximum of *q̃_L_* will lead to minimum of *s̃_T_*, since the minimum of entropy production is directly correlated to minimum of consumed power, which at its turn is linked by Equation (13) to maximum of *q̃_L_* for imposed *COP*.

#### 2.4.2. Imposed Refrigeration Load

The same optimization method is applied in this case, but the terms in equations are expressed function on the imposed non-dimensional refrigeration load, *q̃_L_*, instead of *COP*. Thus, the first constraint equation (C1) of the model, given by Equation (24) in the previous case, becomes:

(46) $${\tilde{q}}_{L}-CTQ_{L,gen}\xi =0,$$

while the second constraint equation (C2) remains the same as in the previous case of imposed *COP*, given by Equation (27).

The objective function *FO* in Equation (21) is either the *COP*, or the non-dimensional entropy production *s̃_T_*. The Lagrangian is computed and a similar system of equations as Equations (35) or (44) is obtained and solved with respect to the same variables *CTQ_L,gen_, θ_H_, θ_L_*:

(47) $$\frac{f^{2}}{\theta_{H}^{2}f’}-\frac{f{g^{\prime }}_{H}}{CTQ_{H,gen}f^{\prime }}=\frac{\xi^{2}}{\theta_{L}^{2}\xi ’}-\frac{\xi {g^{\prime }}_{L}}{CTQ_{L,gen}\xi^{\prime }}\;{\tilde{q}}_{L}-CTQ_{L,gen}\xi =0\;CTQ_{H,gen}\frac{f}{\theta_{H}}+CTQ_{L,gen}\frac{\xi }{\theta_{L}}+g=0\;CTQ_{H,gen}=CTQ_{T,gen}-CTQ_{L,gen}$$

## 3. Results

The optimization method described above leads to a system of non-linear equations that is numerically solved in Matlab by applying Newton-Raphson method. The system parameters set to constant values in the numerical simulation are: total coefficient of heat transfer *CTQ_T,gen_* = 2, non-dimensional low source temperature *θ_LS_* = 0.87 (corresponding to a temperature of the cold source of −5 °C in case of an ambient temperature of 35 °C) and the internal entropy production *s̃_i_* for which different cases have been simulated, as given by Equations (8)–(10), where the “*const*” term was set to a constant value of 0.005. Since the obtained results are similar for the three variation profiles, for the sake of comprehensibility, only the results for constant internal entropy production will be further presented. The other constant values considered correspond to the imposed constraints, such as coefficient of performance *COP* or non-dimensional refrigeration load *q̃_L_*.

The system is solved with respect to the unknown solutions *CTQ_L,gen_*, *θ_L_* and *θ_H_*. The obtained physical solutions allow computing the system performances in terms of *q̃_L_* or *COP* and the other non-dimensional energy rates *q̃_H_*, *w̃*. Also, the total entropy production *s̃_T_* is computed.

The results are presented for the two considered cases, namely imposed *COP* and imposed refrigeration load, respectively. 

### 3.1. Imposed Coefficient of Performance

For the numerical simulation, it’s value has been set to *COP =* 1.5 and for the internal entropy production *s̃_i_* = 0.005 was considered. After solving the system of equations, two physical solutions *θ_L_* and *θ_H_* are obtained. These two solutions correspond to two different operating regimes, namely one of maximum refrigerating load, denoted by subscript 1, and the other leading to minimum total entropy production, denoted by subscript 2 in the figures. As it was analytically proved, the extrema in both regimes correspond to the same optimum values of system parameters. Results are further presented for the case of linear and non-linear heat transfer laws.

#### 3.1.1. Linear Heat Transfer Laws

**Figure 2:**
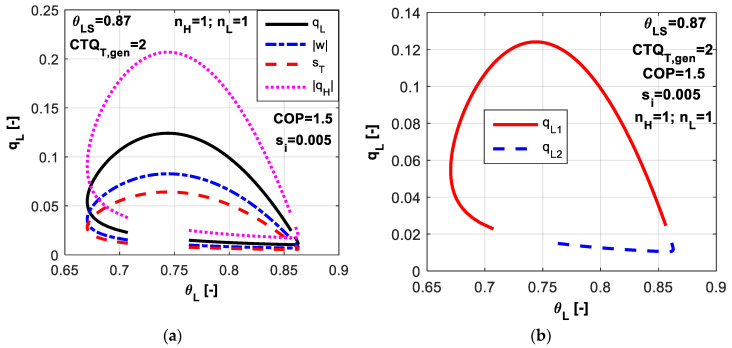
Imposed *COP* and *s̃_i_* constant: (**a**) Non-dimensional energy rates and total entropy production for the two operating regimes; (**b**) Non-dimensional refrigerating load for the two operating regimes.

In Figure 2a, non-dimensional energy rates and total entropy production are presented for the two operating regimes function on the control variable *θ_L_*. The upper part of the plots corresponds to the maximum refrigerating load regime, while the lower one corresponds to the minimum total dissipation regime. One might notice that the most convenient operating parameters are those located in the left part of the maximum values, since here the consumed power and total dissipation are lower for lower cold source temperatures. 

The non-dimensional refrigerating load is plotted versus *θ_L_* generating loop curves as shown in Figure 2b. One chosen value for the refrigerant temperature *θ_L_* might indicate the operation at two refrigerating loads, on the upper part and the lower one respectively

Also, one desired value of the refrigerating load could be associated to a refrigerating temperature closer to the reference one (ambient) as seen in the right-hand-side of the plot, or contrary to a lower temperature value in the left-hand-side. The choice of the operating regime depends on the system settings and the other variables that are behind (*θ_H_*, *CTQ_L,gen_*, etc).

**Figure 3:**
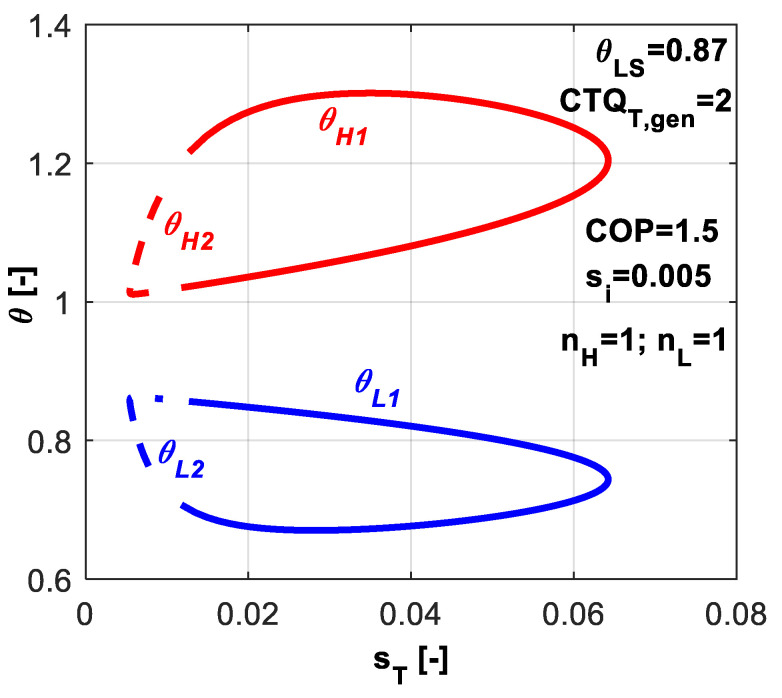
The two solutions of the non-dimensional refrigerant temperatures function on *s̃_T_* for the case with imposed *COP* and *s̃_i_* constant.

Figure 3 presents a *T-s* like diagram. The possible range values are emphasized for the refrigerant temperatures at the hot and cold reservoirs. Also one might notice that the second solution of the system of equations (*θ_L_*_2_, *θ_H_*_2_) corresponds to the lower dissipation rates, but also to the lower energy rates. Thus, the second solution might not be of interest from the operation regime point of view. 

**Figure 4:**
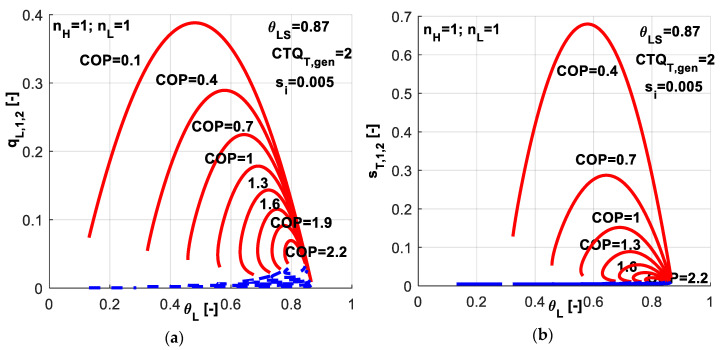
Imposed *COP* and *s̃_i_* constant - sensitivity study with respect to *COP* (**a**) Non-dimensional refrigeration load for the two regimes; (**b**) Non-dimensional total entropy production for the two regimes.

A sensitivity study with respect to *COP* was performed. It revealed the possible range for system variables and the associated performances. For the maximum refrigeration load regime, as higher *COP* values constraint the operation, as lower the refrigerating loads are and the range for refrigerant temperature becomes narrower, as emphasized by red upper curves in Figure 4a. For the minimum total dissipation regime (blue down curves in Figure 4a), the higher *COP* is imposed, the higher refrigerating load is. This particular behavior of the system is explained by the refrigerant temperature variation and heat exchanger inventory distribution in terms of *CTQ_L,gen_* and *CTQ_H,gen_* associated to this solution of the equations system. Also one might notice that when the system operates at an imposed *COP* value, there is a certain refrigerant temperature *θ_L_* leading to a maximum value of the refrigerating load. The corresponding total entropy production is presented in Figure 4b.

**Figure 5:**
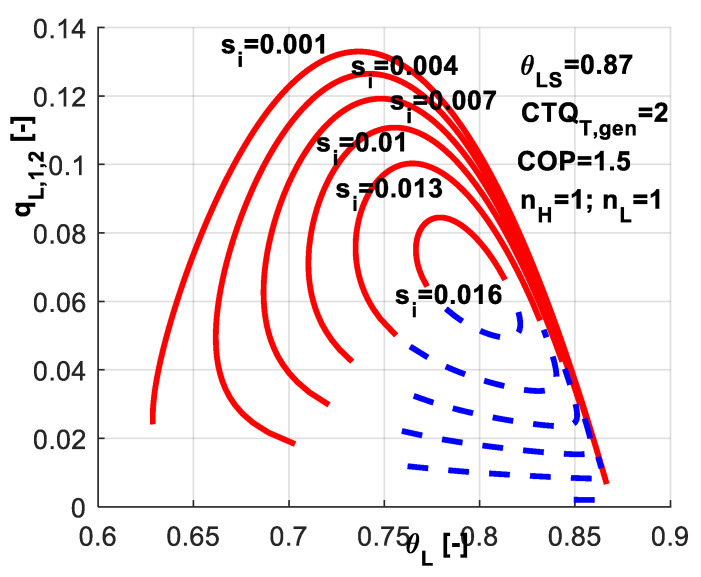
Imposed *COP* and *s̃_i_* constant - sensitivity study with respect to *s̃_i_*. Non-dimensional refrigeration load for the two regimes.

The effect of internal entropy rate *s̃_i_* on system variables and performances was studied, too. Its value is closely related to the built machine. Figure 5 reveals that higher *s̃_i_* values are accompanied by a narrower range for system variables and obviously lower performances. It is interesting to notice that when operating at higher values of the refrigerant temperature *θ_L_*, the effect of *s̃_i_* is more reduced as emphasized by the curves tangent region in the right-hand-side of the plot. Contrary, its effect is very important in the region of lower refrigerant temperatures—left-hand-side of the curves.

**Figure 6:**
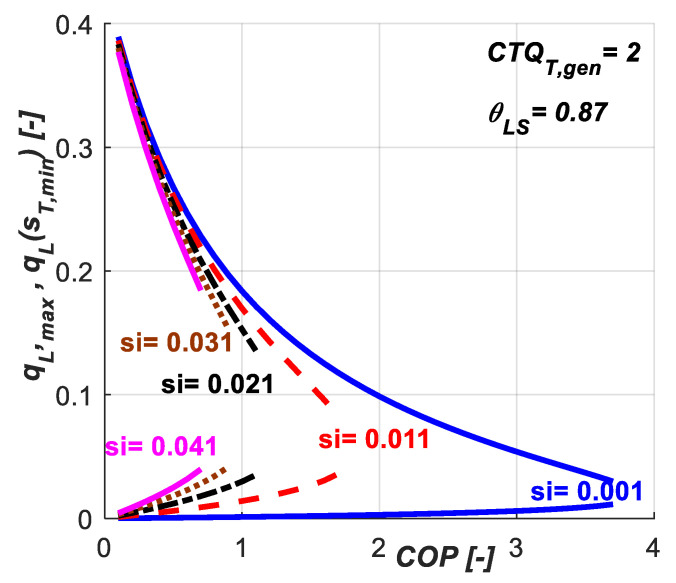
Maximum refrigeration load for a given set of *COP* and *s̃_i_* constant values.

Figure 6 presents the maximum values for the refrigeration load that could be achieved for a given set of *COP* and *s̃_i_* values. It also emphasizes the effect of *s̃_i_* on the possible operating range of the system under these constraints. Lower *s̃_i_* values let the system operates on a wider range of *COP* values. Increasing *s̃_i_* values limits the system performances in terms of achievable *COP* and, diminishes the maximum refrigeration load attainable limit (the upper curves) for a given *COP* value.

#### 3.1.2. Non-Linear Heat Transfer Laws

**Figure 7:**
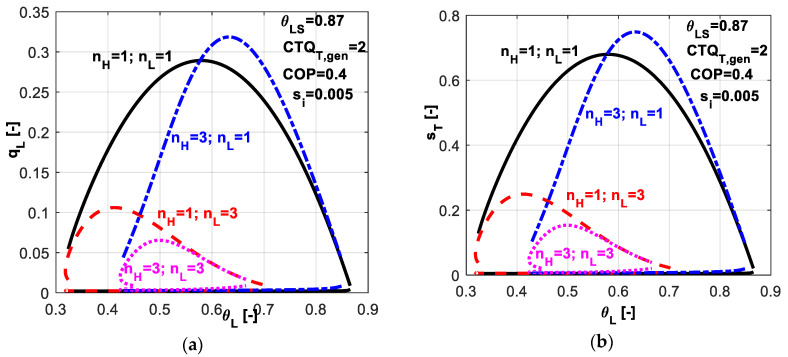
Imposed *COP* and *s̃_i_* constant - sensitivity study with respect to heat transfer laws nature at both reservoirs (**a**) Non-dimensional refrigerating load; (**b**) Non-dimensional total entropy production.

In Figure 7, one might analyze the effect of different nature of heat transfer laws at the source and sink on the non-dimensional refrigerating load (Figure 7a) and total entropy production (Figure 7b). Symmetric and non-symmetric combinations of exponents *n_H_* and *n_L_* have been chosen. The value of 3 corresponds to a phase change process. The nature of the considered heat transfer laws affects the optimum values of system parameters for obtaining maximum system performances and also the magnitude of system performances. 

As revealed by Figure 7a, a phase change process at the cold source (*n_L_* = 3) involves lower refrigerating loads and shifts the maximum achievable one towards lower temperature values; the peak of the curves is shifted towards left with respect to the linear case (*n_H_* = *n_L_* = 1). By constraining the operation at a *COP* value of 0.4 leads to a maximum achievable non-dimensional refrigerating load of about 0.28 in the case of linear heat transfer laws, and 0.06 respectively for the case of phase change processes at both reservoirs. The justification relies on the temperature difference between the two heat sources and the refrigerant that also depends on the heat transfer law, becoming lower for the phase change processes. We also notice that the nature of the heat transfer law influences the range for possible values of the system variables, especially in the case of phase-change processes. 

**Figure 8:**
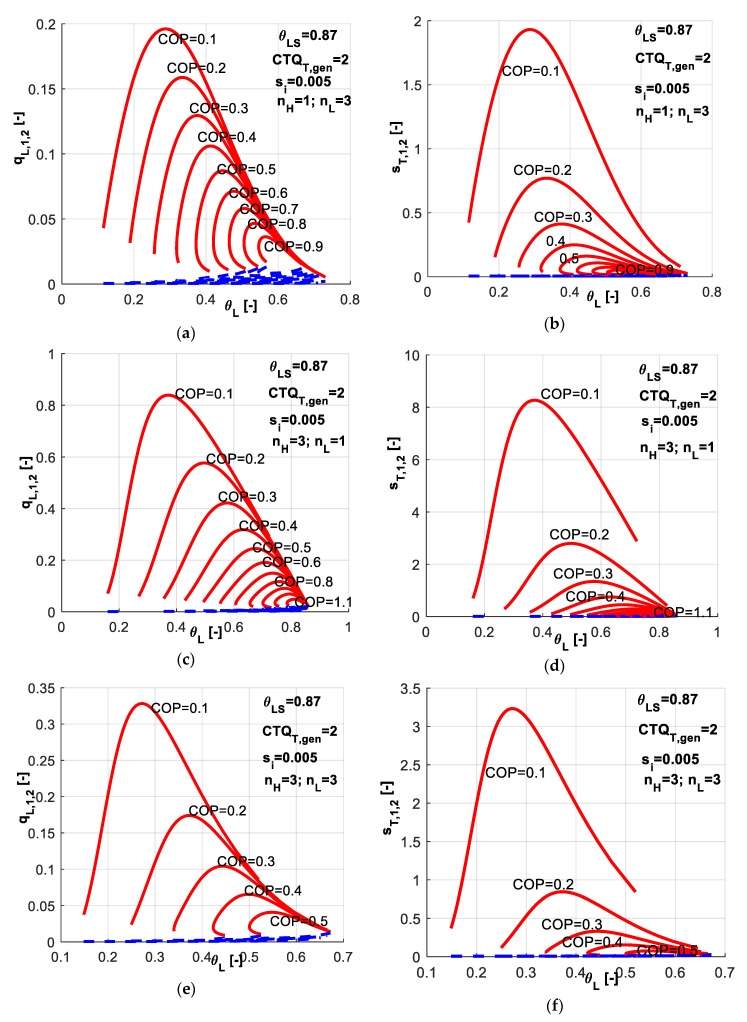
*COP* and *s̃_i_* constant constraints - sensitivity study with respect to heat transfer laws nature at both reservoirs for different combinations (*n_H_*, *n_L_*).

A sensitivity study with respect to *COP* values associated to different combinations of heat transfer laws emphasize the results obtained in Figure 8. One might notice that the different considered combinations of exponents *n_H_* and *n_L_* lead to different possible ranges for *COP* as a constraint. Maintaining the same settings for the machine as previously (*CTQ_T,gen_* = 2, *θ_LS_* = 0.87, *s̃_i_* = 0.005), one observes that:

- in the case of linear heat transfer laws at both reservoirs, the *COP* values could be imposed up to a maximum value of 2.2 (Figure 4);
- when a phase change process in considered at the cold source (Figure 8a,b), the maximum possible *COP* is about 0.9;
- if the phase change process in considered at the hot sink (Figure 8c,d), the maximum possible *COP* is about 1.1;
- while in the case of phase change process at both reservoirs (Figure 8e,f), the *COP* is limited to 0.5. In fact, this is the reason for which a value of 0.4 was considered for *COP* in the simulations presented in Figure 7.

**Figure 9:**
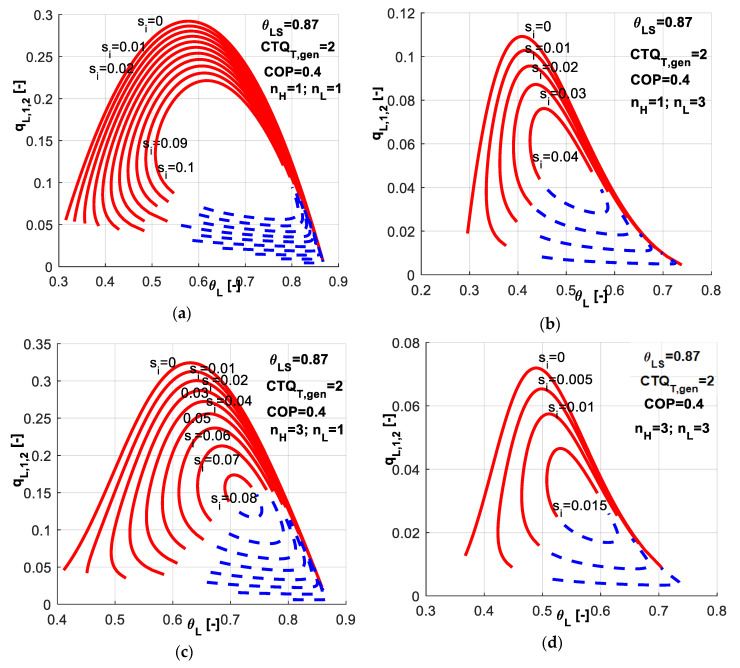
Imposed *COP* and *s̃_i_* constant – non-dimensional refrigerating load for different combinations of heat transfer laws at both reservoirs (*n_H_*, *n_L_*).

Figure 9 presents the combined effect of heat transfer laws and *s̃_i_* constraint values on the non-dimensional refrigerating load and possible range values of refrigerant cold temperature. The first observation regards the *s̃_i_* constraint values. Linear heat transfer laws allow the system to operate at higher internal irreversibilities (Figure 9a) when needed, while a phase change process occurring at a heat source limits the operation to lower internal irreversibilities (Figure 9b,c). The most restrictive case is the one at which phase change processes occur at both reservoirs (Figure 9d), for which the maximum acceptable constraint in terms of *s̃_i_* is about 0.015 under the other given settings (*CTQ_T,gen_*, *θ_LS_*). The second observation refers to the range of achievable *θ_L_* values. The limit case of endoreversible operation (*s̃_i_* = 0) is obviously the most generous in all cases, while the most restrictive one is the case of phase change processes occurring at both reservoirs. For a given value of *s̃_i_* = 0.04 for example, the linear case allows *θ_L_* to be obtained from 0.4 to 0.9 (Figure 9a). When phase change occurs at the cold source (Figure 9b), *θ_L_* is limited to the range 0.42–0.58, while if the phase change occurs at the hot sink (Figure 9c), the range is 0.55–0.81. The four combinations of heat transfer laws (Figure 9d) do not allow at all the system operation in this case.

The above results contribute to the understanding of the system limits in operation under different constraints and allow the engineer to choose the most convenient variables in certain circumstances.

### 3.2. Imposed Refrigeration Load

Similar to the previous case, a numerical simulation was carried out when imposing the refrigeration load instead of *COP*. The considered imposed values for the non-dimensional parameters are *q̃_L_* = 0.1 and *s̃_i_* = 0.005. The other parameters have the same values, namely *θ_LS_* = 0.87 and *CTQ_T,gen_* = 2.

This time the system of equations reveals only one physical solution *θ_L_* and *θ_H_*. This solution corresponds to maximum *COP* operating regime and in the same time to minimum total entropy production one, as proved before (the extrema in both regimes correspond to the same optimum values of system parameters). Results are further presented for the case of linear and non-linear heat transfer laws.

#### 3.2.1. Linear Heat Transfer Laws

**Figure 10:**
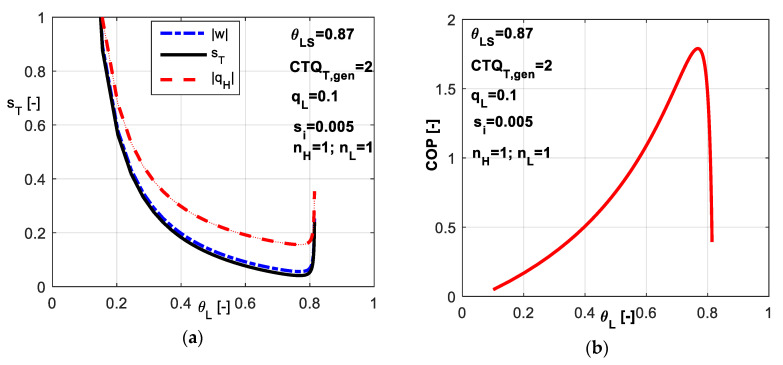
Imposed *q̃_L_* and *s̃_i_* constant (**a**) Non-dimensional energy rates and total entropy production; (**b**) *COP* variation with respect to *θ_L_*.

For the case of linear heat transfer laws, the results are plotted in Figure 10. One might notice a large range for the refrigerant cold non-dimensional temperature *θ_L_* and the existence of an optimum value corresponding to minimum non-dimensional power consumption *w̃* and minimum total entropy production *s̃_T_* (Figure 10a). This optimum value leads also to maximum *COP* as revealed by Figure 10b. Numerically, this point tends to equal distribution of *CTQ* between sources, namely *CTQ_L,gen_* = *CTQ_H,gen_*. The results are similar to the previous case where the imposed value of *COP* leads to an optimum value of *θ_L_* corresponding to maximum *q̃_L_*.

**Figure 11:**
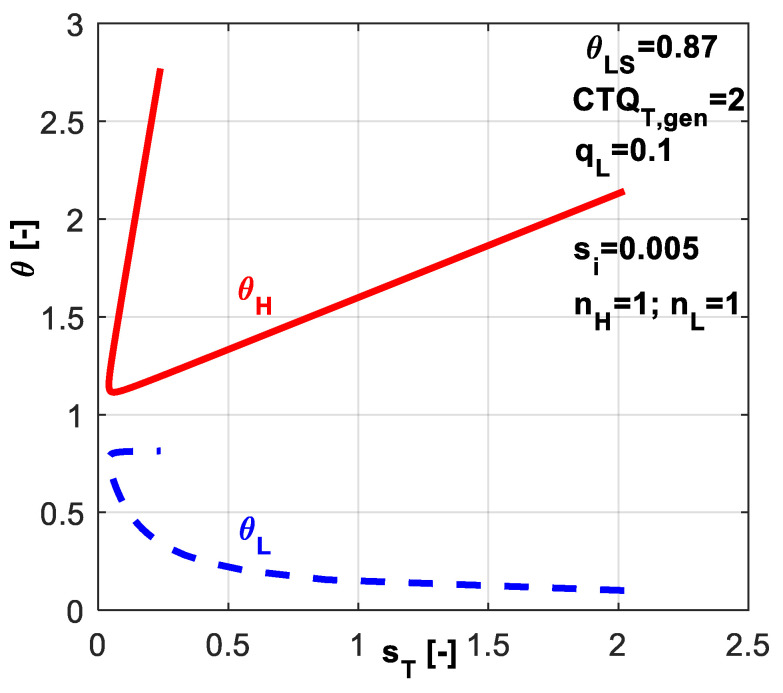
Non-dimensional refrigerant temperatures at hot and cold reservoirs for the case of imposed *q̃_L_* and *s̃_i_* constant.

In Figure 11, the non-dimensional temperatures *θ_L_* and *θ_H_* are plotted versus dimensionless total entropy production *s̃_T_*. Obviously, the cycle operating between minimum difference in refrigerant temperatures is accompanied by the minimum total dissipation (left extrema in Figure 11). As this difference increases, the total dissipation increases.

**Figure 12:**
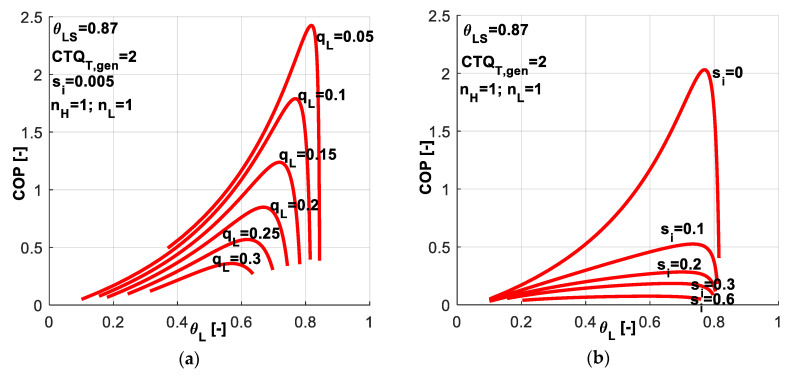
*COP* variation for the case with imposed *q̃_L_* and *s̃_i_* constant (**a**) Sensitivity with respect to dimensionless refrigerating load; (**b**) Sensitivity with respect to dimensionless internal entropy production.

A sensitivity study with respect to the refrigerating load emphasizes the variation range for dimensionless cold refrigerant temperature *θ_L_* limiting the values of *COP* (Figure 12a). The behavior is similar to the one presented for the previous case in Figure 4a limiting the values of *q̃_L_* when imposing *COP*. For each imposed *q̃_L_* value, an optimum *θ_L_* value exists for which the system *COP* is maximum. Higher values of constraint refrigeration load *q̃_L_* diminishe the *COP* values and shift the maximum *COP* towards lower cold refrigerant temperature values *θ_L_*. For the chosen set of system parameters, a dimensionless refrigeration load of 0.3 is the maximum attainable in the range of 0.35–0.62 for *θ_L_* values. The internal entropy production effects are emphasized in Figure 12b. The most important effect is noticed on limiting the *COP* values, rather than limiting *θ_L_*.

**Figure 13:**
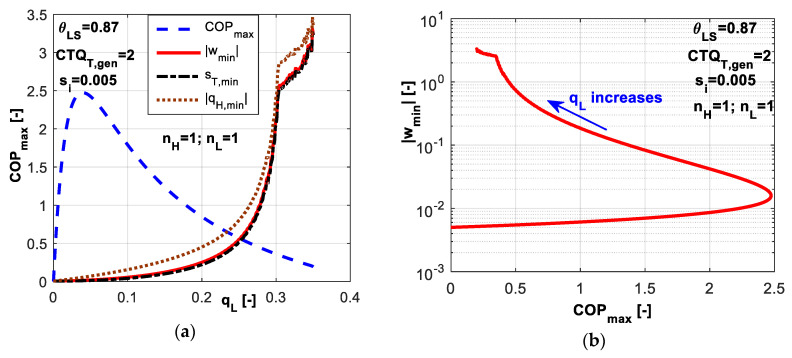
*q̃_L_* influence: (**a**) Dimensionless energy rates and *COP* variation–sensitivity with respect to *q̃_L_*; (**b**) Dimensionless consumed power versus maximum achievable *COP*.

Figure 13 presents the variation of maximum *COP* values and the corresponding minimum energy rates and total entropy dissipation with dimensionless refrigerating load. A maximum value among the *COP* maxima is obtained for *q̃_L_* = 0.04 in the given conditions. These types of plots could be used to set the system operating regimes for maximum performances under some imposed constraint. Figure 13b emphasizes the variation of dimensionless consumed mechanical power with maximum *COP*, at logarithmic scale, for increasing *q̃_L_* values. This plot puts into evidence that the chosen operating regime is a trade-off between *COP* and refrigerating load *q̃_L_*, for high value of *q̃_L_*. The lower part of the curve corresponds to low values of *q̃_L_* and *w̃*, and the value of maximum *COP* appears as a transition point.

#### 3.2.2. Non-Linear Heat Transfer Laws

**Figure 14:**
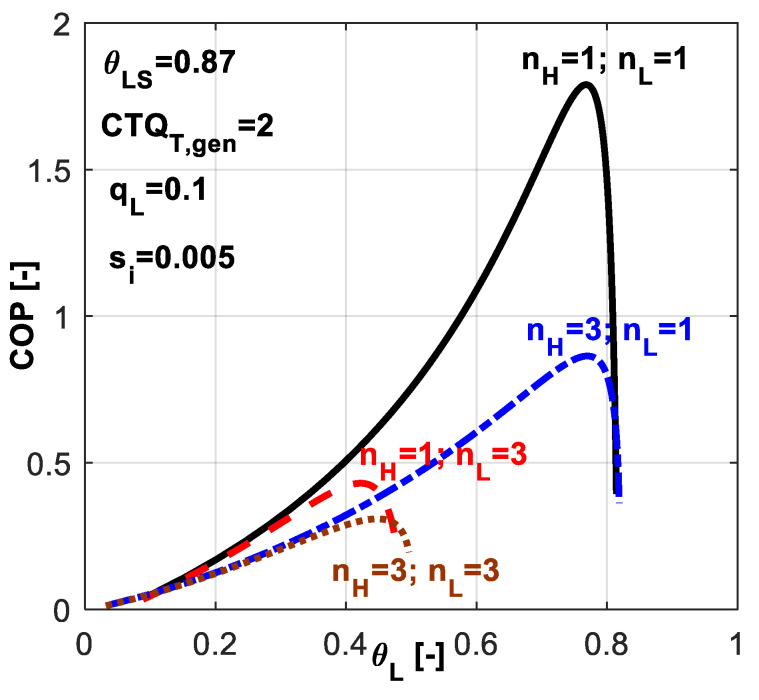
*COP* variation for the case of imposed *q̃_L_* and *s̃_i_* constant - sensitivity study with respect to heat transfer laws nature at the source and sink.

The effect of different combinations of heat transfer laws on the system *COP* is emphasized in Figure 14. The most favorable combination from *COP* point of view is the case of linear heat transfer laws at both sides, while the most constrainable is the one considering phase change processes at both sides. These two combinations constraint the *COP* values and also the range for allowable *θ_L_* values.

**Figure 15:**
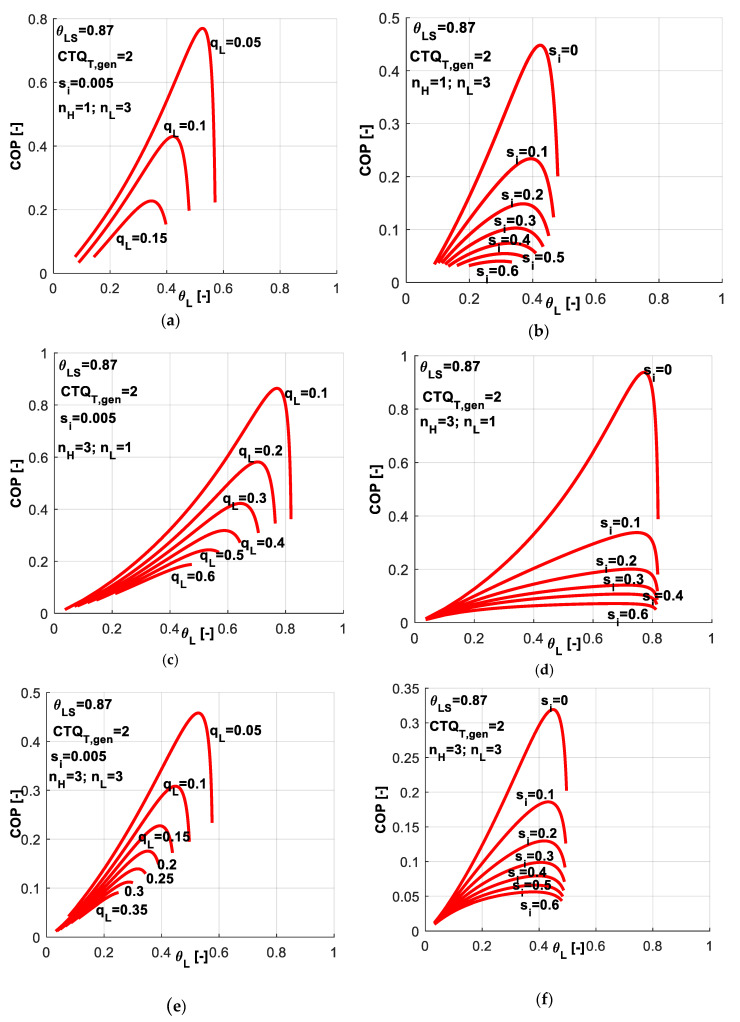
*COP* variation for the case of imposed *q̃_L_* and *s̃_i_* constant, for different combinations of heat transfer laws - sensitivity study with respect to *q̃_L_* and *s̃_i_*.

Figure 15 presents a sensitivity study with respect to *q̃_L_* and *s̃_i_* values associated to different combinations of heat transfer laws. This study is similar to the one presented in Figure 8 when imposing *COP* instead of *q̃_L_*. One might notice that the different considered combinations of exponents *n_H_* and *n_L_* lead to different possible ranges for *q̃_L_* as a constraint and for *θ_L_* as control variable. Maintaining the same settings for the machine in all cases (*CTQ_T,gen_* = 2, *θ_LS_* = 0.87, *s̃_i_* = 0.005), one observes that:

- in the case of linear heat transfer laws at both reservoirs, the *q̃_L_* values could be imposed up to a maximum value of about 0.3 (Figure 12a); the limits in *θ_L_* are quite large and the *θ_L_* values are naturally decreasing as *q̃_L_* decreases;
- when a phase change process in considered at the cold source (Figure 15a,b), the maximum possible *q̃_L_* is about 0.15, half of the above-mentioned value; also the limits for *θ_L_* values are much reduced;
- if the phase change process in considered at the hot sink (Figure 15c,d), the maximum possible *q̃_L_* is about 0.6, so twice with respect to the linear case; the limits for *θ_L_* values are comparable to those obtained for the linear case;
- while in the case of phase change process at both reservoirs (Figure 15e,f), the *q̃_L_* is limited to 0.35, and thus the effects of the two phase change processes are cancelling each other when comparing to the linear laws; *θ_L_* values are more reduced in this case.

**Figure 16:**
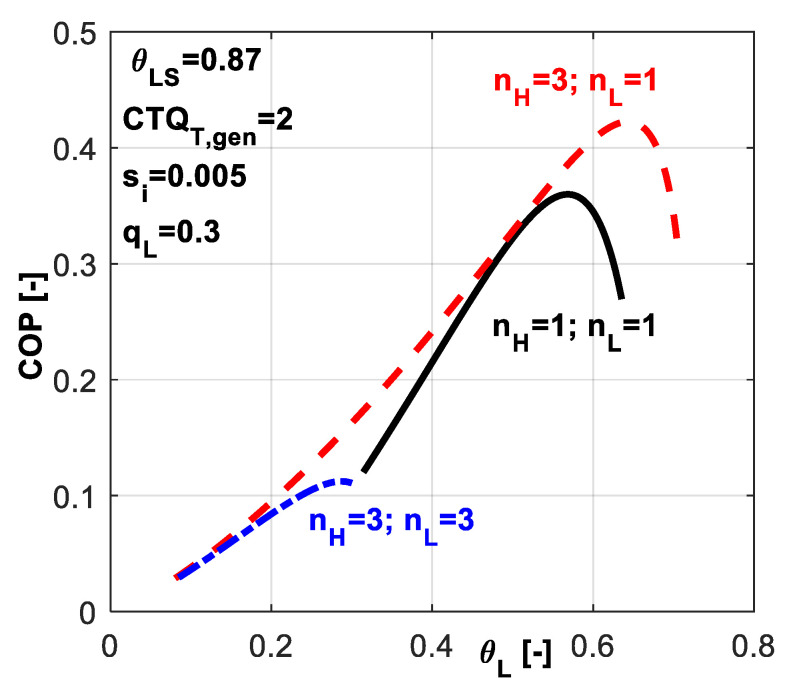
*COP* variation for the case of imposed *q̃_L_* and *s̃_i_* constant - sensitivity study with respect to heat transfer laws nature at the reservoirs.

One might deduce from these results that when a system is supposed to undergo a deep cooling process, the nature of the heat transfer law is very important. In this regard, Figure 16 reveals the effect of chosen heat transfer law for such applications, when the required dimensionless refrigerating load is more important, here *q̃_L_* = 0.3.

One might notice that for this application, the best alternative is to choose a linear heat transfer law at the sink and a phase change process at the hot source (*n_H_* = 3, *n_L_* = 1). This combination leads to maximum *COP* for a required cold temperature and also it allows the system operation over a wider range of *θ_L_* values. From Figure 15c one may also notice that this combination ensures the system operation at heavier refrigerating loads.

## 4. Conclusions and Perspectives

A general model for the study and optimization of irreversible refrigeration Carnot-like machines was presented. The study started with machines optimization under different constraints and ended with important aspects related to the intrinsic phenomena affecting the systems operation. 

The results confirm that the First and Second Law of Thermodynamics are very useful tools in optimization problems under constraints.

The system limits in operation under different constraints were emphasized from the point of view of possible values for system control variables (such as *θ_L_*), for imposed constraint (*COP* or *q̃_L_*) and achievable performances (*q̃_L_* or *COP*, *s̃_T_*, other dimensionless energy rates). Moreover, the results are useful in deciding the most convenient values and heat transfer laws in particular circumstances and for specific applications. 

The above results contribute to the understanding of the system limits in operation under different constraints and allow the engineer to choose the most convenient variables in given circumstances. 

Optimal variables are found for the best performances that the system could achieve under specified constraints. Also the limits of the system operation are determined.

Further development of the model for other constraints (imposed *w̃* or *q̃_H_*) is in progress. Also, a comparison of the direct and reverse machine models and results are under consideration. It seems very promising and gives a new perspective on their optimization through a unitary approach.

## List of Symbols

*A*	heat transfer area, [m^2^]
*COP*	coefficient of performance, [-]
*CTQ_gen_*	generalized number of heat transfer units, [-]
*f, g*	functions defined in the model
*K_gen_*	generalized conductance, [W K^−n^]
$\dot{m}c_{p}$	heat rate capacity, [W K^−1^]
*n*	exponent of the heat transfer law, [-]
$\dot{Q}$	heat transfer rate, [W]
$\tilde{q}$	dimensionless heat transfer rate, [-]
$\dot{S}$	entropy production, [W K^−1^]
$\tilde{s}$	dimensionless entropy production, [-]
*T*	temperature, [K]
*U*	overall heat transfer coefficient, [W m^−2^ K^−1^]
$\dot{W}$	mechanical power, [W]
*w̃*	dimensionless mechanical power, [-]
*Greek Symbols*	
*λ*	Lagrange multiplier
*θ*	non-dimensional temperature
*ξ*	function defined in the model
*Subscript*	
*gen*	generalized
*H*	working fluid, at the machine hot temperature side
*HS*	source, at the machine hot temperature side
*i*	internal
*L*	working fluid, at the machine low temperature side
*LS*	source, at the machine low temperature side
*T*	total
*Abbreviations*	
*C*	constraint
*FO*	objective function
*L*	Lagrangian function
